# Clinically approved immunotoxins targeting hematological cancers: “the best of both worlds”

**DOI:** 10.3389/fphar.2025.1569502

**Published:** 2025-10-17

**Authors:** Yasmine Rashad, Eckhard U. Alt, Reza Izadpanah, Xuebin Qin, Stephen E. Braun

**Affiliations:** ^1^ Applied Stem Cell Laboratory, Department of Medicine, Heart and Vascular Institute, Tulane University School of Medicine, New Orleans, LA, United States; ^2^ Department of Immunology and Microbiology, Tulane University School of Medicine, New Orleans, LA, United States; ^3^ Division of Comparative Pathology, Tulane National Primate Research Center, Covington, LA, United States; ^4^ Division of Immunology, Tulane National Primate Research Center, Covington, LA, United States; ^5^ Department of Pharmacology, Tulane University School of Medicine, New Orleans, LA, United States

**Keywords:** hematological malignancies, cancer immunotherapy, recombinant immunotoxins, diphtheria toxin, *Pseudomonas* exotoxin, moxetumomab pasudotox, tagraxofusp, E7777

## Abstract

Hematological malignancies contribute significantly to the overall cancer burden. Certain subtypes, such as hairy cell leukemia (HCL), are chronic and characterized by residual disease after first-line therapy, while others, such as blastic plasmacytoid dendritic cell neoplasm (BPDCN), are aggressive and associated with poor prognosis. Although cornerstone interventions such as radiation and chemotherapy are efficiently used to treat some malignant blood neoplasms, these treatments are often limited by resistance, relapse, lack of enduring disease-free survival/complete remission, and systemic toxicity. Immunotoxins were developed to improve tumor targeting and have evolved into recombinant immunotoxins (RITs). These novel bioengineered chimeras genetically combine potent cytotoxins with targeted binding domains. In this review, we analyze three FDA-approved RITs, namely, moxetumomab pasudotox, tagraxofusp, and denileukin diftitox, that utilize bacterial toxins from *Pseudomonas* and *Corynebacterium diphtheriae* to treat refractory/relapsed (R/R) HCL, BPDCN, and adult R/R cutaneous T-cell lymphoma (CTCL), respectively. We reviewed their comprehensive safety profiles, describe complications associated with these fusion proteins, and, finally, discuss potential risk management strategies that may enhance their clinical outcomes. Overall, RITs have demonstrated efficacy, and researchers continue to extend these findings to other indications.

## Introduction

According to the National Cancer Institute registry, in 2023, hematological malignancies accounted for ∼9.4% of new cancer cases and were responsible for 9.4% of reported cancer deaths within the United States. The 5-year (2013–2019) estimated survival rate for leukemia was 66.7% (Cancer Statistics, 2024). Although traditional cancer therapies have advanced over the past decades, detectable minimal residual disease (MRD) post-treatment, health vulnerability in old age, systemic adverse effects including bystander cell/tissue toxicity, relapse, and chemo-resistance continue to pose major challenges in blood cancer management. This emphasizes the quest for a highly specific curing therapy, like a “magic bullet.”

Chemo-monotherapy is generally effective in some hematological malignancies, such as diffuse large B-cell lymphoma, and some T-cell malignancies, such as acute leukemia. However, chemo-resistance still constrains this approach. For example, acute myeloid leukemia (AML) is characterized by several functional chemo-resistance mechanisms, which include drug efflux pumps (Yeung and Radich, 2017). Accordingly, immunotherapy has been introduced for cancer treatment and aims to enhance the host’s immune system in combating malignancies (Farkona et al., 2016).

Recombinant immunotoxins (RITs), a type of immunotherapy, have shown promise in compensating for these unmet qualities of traditional systemic treatments. The recombinant technology provides consistency in combining their two subunits, a tumor-specific targeting moiety genetically fused to a fast-acting modified cytotoxin (Allahyari et al., 2017). Potential RITs are highly stable (protein stability of the chimeric fusion is tested at body temperature), bind with high affinity to tumor-specific antigens (TSAs), and can effectively translocate into the cytosol and become cytotoxic (FitzGerald et al., 2004). These properties of RITs could limit systemic toxicity and drug resistance experienced with conventional modalities while eradicating MRD to achieve a complete response (CR) more rapidly. Thus, they improve the efficiency of targeting residual disease in adult populations and provide other strategies for patients without the option of hematopoietic stem-cell transplant (HSCT).

Hairy cell leukemia (HCL) is an example of a chronic B-cell leukemia characterized by cytopenia (Morton et al., 2006). Purine nucleoside analog (PNA) chemotherapy, using either cladribine or pentostatin, is the current standard of care for *de novo* HCL. Else et al., (2009) reported a CR of 76% in patients treated with cladribine and 82% in those treated with pentostatin in an assessment of 233 patients with HCL. However, these regimens often fail to induce a durable disease-free plateau. Refractory/relapsing disease was also found by Else et al., (2009) at 38% relapse with cladribine and 44% with pentostatin. Despite a high initial CR rate with PNA chemotherapy, MRD is often detected in HCL patients post-treatment. Getta et al. (2015) identified MRD in 27%–50% of a CR patient sample. Second-line therapy is indicated based on CR sustainability and includes retreatment with PNAs or different combinations of chemo-immunotherapy (rituximab with PNA or bendamustine) (Maitre et al., 2019). However, subsequent PNA courses eventually decline the response rates, accumulate toxicity (Kreitman and Pastan, 2020; Seymour et al., 1994; Seymour et al., 1997; Tadmor, 2011), and increase susceptibility to secondary malignancies (Morton et al., 2006; Getta et al., 2016). The risk of infection is also higher as both these PNAs are immunosuppressive, especially cladribine (Kreitman et al., 2021). As a result, the FDA approved moxetumomab pasudotox (MP) for managing refractory/relapsed (R/R) HCL (FDA, 2020). Compellingly, MP may also play a role in eradicating MRD in patients with HCL and may potentially be associated with a reduced relapse rate.

Another hematological malignancy, blastic plasmacytoid dendritic cell neoplasm (BPDCN), was originally categorized by WHO under AML before they identified it as its own individual disease in a 2016 revision (Arber et al., 2016). BPDCN’s rare incidence and unclear understanding of its biology challenged the establishment of a specific standard of care. BPDCN is an aggressive cancer with an extremely low incidence rate of <0.5% (Shi and Wang, 2014; Rauh et al., 2012; Yu et al., 2014) that mainly affects adults (∼60–70 years). It is less commonly observed in the pediatric population, which has historically shown great outcomes with existing leukemia/lymphoma-based treatment protocols. Unlike younger patients, multiple studies report poorer prognosis in the older population (Shimony et al., 2025). Kharfan-Dabaja et al. (2013) found a median estimated survival of <18 months with chemotherapy in affected adults. Treating BPDCN with chemotherapy resulted in an approximate average of 21.5% early mortality (Pemmaraju et al., 2019). As immunocompetency declines with age, patients likely cannot tolerate immunosuppressive chemotherapy, which could affect their chances of undergoing further treatments, such as HSCT and additional pre-conditioning requirements (Tay et al., 2019). The disease presents with cutaneous lesions but can also occur without dermatological involvement (Rauh et al., 2012; Yu et al., 2014). In these cases, systemic treatment/conditioning with chemotherapy/radiation may be only palliative, which has prompted investigations into the antineoplastic activity of RITs against BPDCN. Currently, tagraxofusp is the only specific treatment for BPDCN approved by the FDA and the European Medicines Agency (Shimony et al., 2025).

The tumor behavior of some mature T-cell lymphomas (TCL), such as cutaneous T-cell lymphoma (CTCL) and peripheral TCL, also shows frequent relapse, refractory disease, and poor prognosis in the later stages. For local manifestations, treatment could include topical creams or light therapy. For extensive skin involvement, current protocols include systemic chemotherapy, such as cyclophosphamide, doxorubicin, vincristine, and prednisone; extracorporeal photopheresis; or radiation. However, the low reported long-term survival average of 25%, along with disease relapse post-HSCT, has encouraged the pursuit of alternative second-line therapies (Hamadani et al., 2014; Khan and Sawas, 2019). Denileukin diftitox (DD), an RIT preparation now reformulated as E7777 (Lymphir), is FDA-approved for treating adult R/R CTCL (Kawai et al., 2021).

In this study, we discuss these three FDA-approved RITs against hematological malignancies—moxetumomab pasudotox (MP), tagraxofusp, and denileukin diftitox (DD)—with an emphasis on the bacterial toxins utilized in these agents. This review encompasses these chimeras’ limitations, safety profiles, and suggested improvement capacities. Increased targeting and specific toxicity would be “the best of both worlds.”

## IT design and development

### Toxin moiety: characterization of bacterial-based ITs

William Coley established the preliminary form of immunotherapy using bacterial components for inoperable cancer treatment in the late 1890s. He cured sarcomas with “Coley’s toxins”—a mixture of *Streptococcus pyogenes*, *Serratia marcescens*, and bacterial products (McCarthy, 2006). In the late 1800s and early 1900s, Paul Ehrlich developed the concepts of tumor-specific markers and, later, the concept of specific drugs that target diseased tissue and used the term “magic bullets.” He suggested that aberrant cells were common but maintained by host factors. Advances in the understanding of modern immunity have provided some insights into host defenses and changes in cell-surface expression, forming the foundation for tumor-specific targets (Valent et al., 2016).

Subsequently, the immunotoxin technology evolved over several generations, starting from a few research groups in the 1970s and 1980s using different toxins (Moolten et al., 1975; Krolick et al., 1982). The first generation used whole toxins conjugated to antibodies. Then, the second generation eliminated unessential portions of the toxin to mitigate the vascular-leak adverse reactions. Finally, RITs are the latest generation using recombinant DNA to fuse the binding and toxin domains (Allahyari et al., 2017).

RITs refer to genetically fusing the sequences of the antibody’s cell-binding fragments to sequences of the modified toxin. This construct allows further alterations to increase binding affinity or cytotoxic activity (Greer et al., 2018; Matthey et al., 2000). The two most commonly used toxins in RIT are *Pseudomonas* exotoxin (PE) and diphtheria toxin (DT). They belong to the same group of ADP-ribosylating toxins and originate from the AB toxin family, consisting of catalytic (A) and binding (B) counterparts. PE and DT are favorable because of their limited non-specific toxicity compared to that of other toxins (including plant toxins). They are also easily cloned and expressed, which makes them cost-effective molecules (Mazor and Pastan, 2020).

Both PE and DT have single-chain polypeptide structures with efficient cytotoxic activity using an analogous mechanism. Each toxin inhibits the function of EF-2 translocase, an elongation factor in protein synthesis, by transferring the ADP-ribose from NAD^+^ to the diphthamide residue on EF-2, which then blocks translation and induces apoptosis (Mei et al., 2019; Michalska and Wolf, 2015; Weldon and Pastan, 2011; Zdanovsky et al., 1993). PE and DT follow receptor-mediated endocytosis (Zdanovsky et al., 1993; Shafiee et al., 2019); however, they follow distinct cytosolic translocation pathways. PE (including the PE38 variant used in RIT) requires assistance with cytoplasm translocation using the retrograde pathway from the ER to the cytosol through the ER’s protein channels. In contrast, DT bypasses the ER and moves directly through the endosomal membrane (in a pH-dependent manner) into the cytosol (Michael Lord and Roberts, 1998). Additionally, the structural and enzymatic domains for each toxin are in different orientations (Srivastava and Luqman, 2015).

### PE in the RIT structure

The native PE precursor molecule is 638 amino-acids (aa), of which the 25 aa signal sequence is cleaved during secretion (Allured et al., 1986). A mature PE molecule is a single polypeptide 613 aa chain: domain Ia (1 aa–252 aa) binds the receptor on target cells, domain II (253 aa–364 aa) enables cytosol translocation, and domain Ib (365 aa–404 aa), along with III (405 aa–613 aa), catalyzes ADP-ribosyl transferase activity.

**FIGURE 1:**
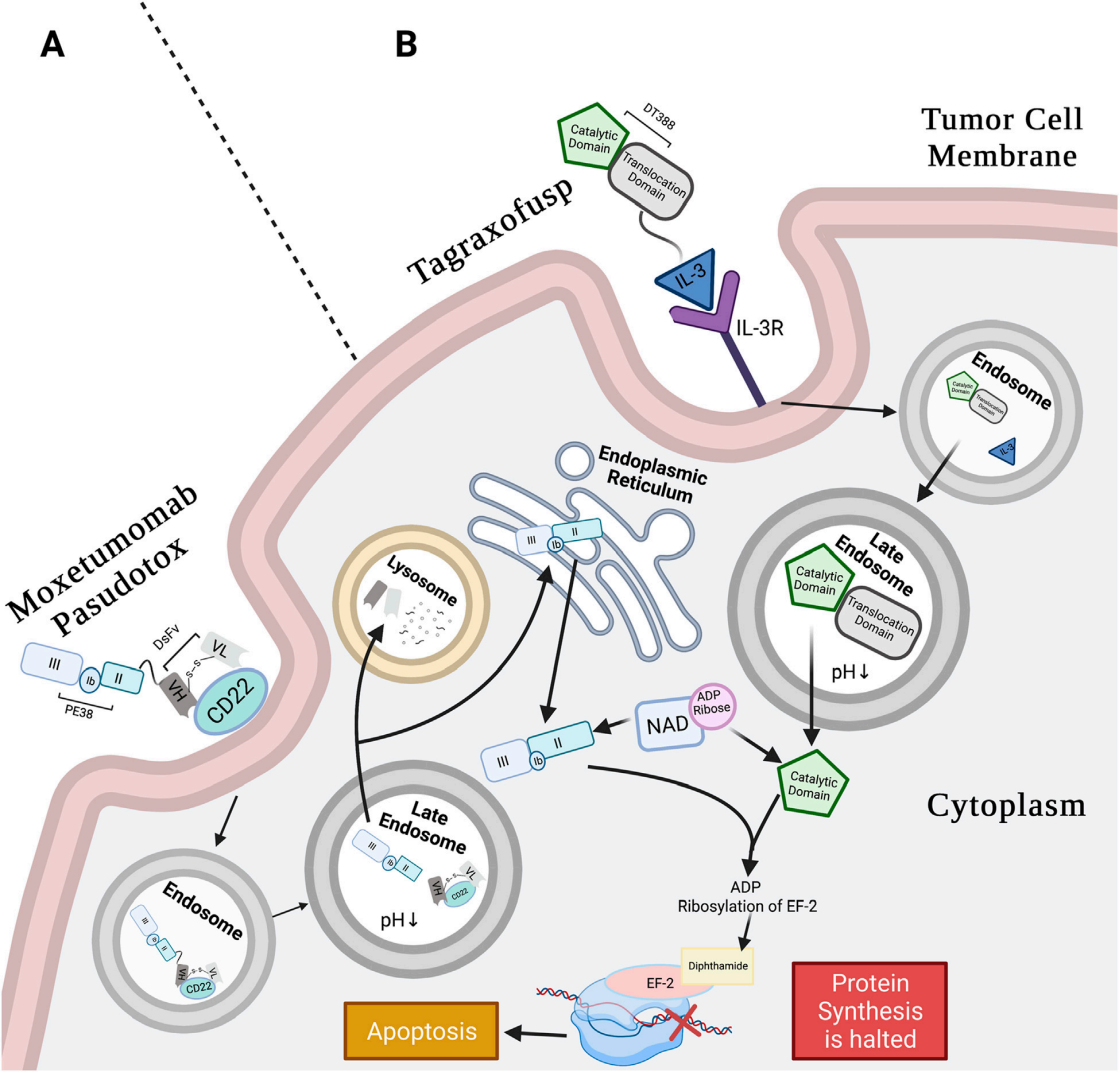
Moxetumomab pasudotox’s and tagraxofusp’s mechanism of action (MoA). **(A)** Moxetumomab pasudotox MoA: anti-CD22 DsFv of RIT binds CD22 on B-tumor cells. The whole conjugate is internalized in clathrin pits via receptor-mediated endocytosis. Pit containing conjugate buds into early endosome and then late endosome, where low pH separates the PE38 from the dsFv, and furin cleavage releases the catalytic payload. Next, the dsFv is degraded in the lysosome, and PE38 moves retrogradely to the ER, likely using the REDL motif and KDEL receptor pathway. The catalytic PE38 payload enters the ER and then the cytosol, where it catalyzes ADP-ribosylation of EF2 diphthamide. This inactivates EF-2 protein synthesis function, leading to apoptosis (Weldon and Pastan, 2011; Kreitman and Pastan, 2011; Abou Dalle and Ravandi, 2019; Pastan et al., 2006). **(B)** Tagraxofusp MoA: IL-3 subunit of tagraxofusp binds with high affinity to CD123 (α-chain of IL-3R) that is highly expressed on BPDCN cell surface and is also internalized through endocytosis. The drug relies on the late endosome for acidification to weaken bonds between the T- and C-domains. Unlike PE38, the C-domain of the RIT is directly translocated to the cytosol to catalyze NAD^+^ by transferring the ADP-ribose molecule to diphthamide on EF-2, interfering with EF-2 protein synthesis activity, and inducing apoptosis (Shafiee et al., 2019; Alkharabsheh and Frankel, 2019). Created in BioRender. Rashad, Y. (2025) https://BioRender.com/p1vrc3a.

Generally, in PE-mediated RITs, the recombinant binding domain genetically replaces the minor domain Ia, while domains III, II, and a subunit of Ib are preserved in the chimeric molecule (Hansen et al., 2010). Removal of domain Ia resulted in PE40, a truncated (40 kDa) PE (Wolf and Elsasser-Beile, 2009; Pastan et al., 1992). To further reduce immunogenicity, more residues were deleted from domain Ib, resulting in PE38, a 38 kDa toxin. The PE38 form of *Pseudomonas* protein toxin A has been commonly used in RITs; one such example is MP (Kreitman et al., 2018a). The intracellular processing of MP, schematically illustrated in Figure 1A, is similar after binding, presumptively using the KDEL receptor pathway.

### PE cytotoxic pathway

Native PE is cleaved at the 613 aa in the extracellular environment; this is hypothesized to be carried out by host plasma carboxypeptidases, excising lysine and resulting in a motif change to REDL. The REDL motif enables the toxin to bind KDEL receptors at the Golgi apparatus during intracellular trafficking. PE binds at domain Ia to a low-density lipid-related protein (LRP), which is also known as CD91 or the α2-macroglobulin receptor, and is internalized by receptor-mediated endocytosis (Wolf and Elsasser-Beile, 2009).

Intracellularly, two pathways are available for PE to reach the ER, namely, the KDEL receptor-mediated pathway and the lipid-dependent sorting pathway (Wolf and Elsasser-Beile, 2009; Pastan et al., 1992). Using the KDEL receptor-mediated pathway, the PE bound to CD91 translocates intracellularly via clathrin-coated pits. As the complex reaches the endosome, low pH induces the dissociation of LRP from PE, and a conformational change exposes the furin protease motif site (in domain II). PE is cleaved into two units that are still attached by disulfide bonds. These bonds are reduced in the late endosome, resulting in 27 kDa and 37 kDa fragments containing domains II, Ib, and III. The 37 kDa fragment reaches the trans-Golgi-network (TGN), where the REDL motif of PE binds to the KDEL receptor and the toxin is translocated to the ER in retrograde movement. In the cytosol, the 37 kDa fragment catalyzes the ADP-ribosylation by binding NAD^+^ and cleaving the bond between the nicotinamide and ribose of the NAD^+^ molecule. This stimulates ADP-ribose transfer to diphthamide, which is a post-translationally modified histidine only found on EF-2. Since the main function of EF-2 is to translocate mRNA across the ribosome, inhibition of EF-2 activity halts protein synthesis, induces cell-cycle arrest, and subsequently induces apoptosis.

### DT in the RIT structure

DT was discovered by Yersin and Roux in 1888 and has been one of the most investigated bacterial toxins. Its potent cytotoxic properties can induce cell death with as low as a single molecule of DT (Yamaizumi et al., 1978; Kolybo et al., 2013) or a minimal lethal dose of <0.1 µg per kg of body weight in humans (Kolybo et al., 2013). It is a single polypeptide Y-shaped chain consisting of three domains within two subunits totaling 535 aa residues. The C-domain (1 aa–193 aa) resides within the A subunit, while the T-domain (201 aa–384 aa) and the R-domain (385 aa–455 aa) are located within the B subunit. The C-domain is responsible for catalyzing the ADP-ribosylation of EF-2, the T-domain is the transmembrane subunit, and the R-domain is the receptor-binding subunit (Mazor and Pastan, 2020). Only lysogenic corynephage-infected *Corynebacterium diphtheriae* secrete DT or are toxicogenic as the phage carries the *tox* gene that encodes DT (John, 1996).

When using DT in a genetically engineered RIT, the R-domain is substituted with a smaller and more stable targeting molecule, while the functional activity of the T- and C-domains is retained. Once inside the cell, RITs employ the same cytotoxic mechanism as native DT (Shafiee et al., 2019). This is shown in Figure 1B, which shows the pathway of the FDA-approved tagraxofusp, a DT-mediated RIT.

### DT cytotoxic pathway

The native DT cytotoxic pathway begins with the R-domain of DT binding to type 1 transmembrane protein pro-heparin-binding epidermal growth factor (pro-HB-EGF) and CD9 on the mammalian cell surface (Srivastava and Luqman, 2015; Kolybo et al., 2013). Upon binding, cell surface proteolytic enzymes cleave the polypeptide bond between the C- and T-domains, which allows for internalization by endocytosis (some DT particles escape surface furin-mediated cleavage but are later cleaved intracellularly instead by furins in the early endosome lumen). Translocation of the C- and T-domains across the plasma membrane and into the endosome takes place in enclosed clathrin-coated pits. At this point, the C- and T-domains are still linked together by a disulfide bond in the endosome. Upon acidification and the reduction of the disulfide bond, conformational changes in the T-domain cause the hydrophobic regions to project through the endosomal membrane and channel the C-domain into the cytosol, where the enzymatic domain’s toxic function is activated. Acidification is crucial for DT intracellular translocation (Antignani and Fitzgerald, 2013). The C-domain binds NAD^+^ in the cytosol and transfers ADP-ribose of the NAD to diphthamide on EF-2. This ADP-ribosylation halts EF-2 activity, causing cell death (Shafiee et al., 2019; Srivastava and Luqman, 2015).

### Targeting moiety

Candidate targeting molecules (i.e., growth factors, cytokines, monoclonal antibodies (mAbs), or fragments of mAbs) should be able to bind with high affinity to the designated (malignant) mammalian cells and effectively deliver the attached toxin. Targeted TSA should be abundantly and homogenously expressed on the tumor cell surface at moderate-to-high density to avoid off-target or competitive binding. It should also be anchored to the plasma membrane, instead of being freely available, to ensure that the toxins bind the tumor cell and reach the cytoplasm (Brown et al., 2008). The technology to retain the antigen-binding fragment (Fv) and eliminate the constant region (Fc) from the targeting mAb facilitates rapid clearance of the RIT from the circulation. This helps reduce the negative effect of unwanted interactions between the hybrid conjugate and vital cells (Mansfield et al., 1997). Ligand binding to their receptors has also demonstrated a potent ability to deliver the RIT molecule with high specificity to tumor cells. Currently, the investigated ligands include interleukins (IL) 2, IL-3, and granulocyte-macrophage colony-stimulating factor (GM-CSF) (Kim et al., 2020).

After the receptor–ligand binding, internalization is crucial for proper toxin uptake. The DT and PE exploit the receptor-mediated endocytosis pathway to cross the plasma membrane in clathrin-coated vesicles and ultimately induce cytotoxicity through the inhibition of EF-2 activity (Michalska and Wolf, 2015; Murphy, 2011).

### Linker

Linkers are vital components in RITs. They play a structural role in attaching the toxin to the binding molecule. Biological linkers enhance the stability and expression of the RIT-targeting domains, particularly those derived from mammalian proteins (i.e., cytokines and growth factors) (Amet et al., 2009; Chen et al., 2013). Chen et al. (2013) reported three main empirical linker classifications in drug delivery applications: rigid, flexible, and cleavable linkers. In protein fusion biologics, linkers can be helical or non-helical (George and Heringa, 2002). Peptide linkers also serve as furin cleavage sites. In toxins, these proteolytic cleaving sites are necessary to activate the catalytic domains’ cytotoxic activity (Weldon et al., 2015). Conceivably, a robust linker would preserve the functions of both domains (binding and toxicity) without compromising drug potency.

## Clinical pearls of immunotoxins: efficacy and toxic effects

To date, there are three FDA-approved RITs indicated for hematological malignancies: tagraxofusp, MP, and DD. Table 1 summarizes some of the history of bacterial-based toxins and their clinical status.

**TABLE 1:** Bacterial-based immunotoxins targeting hematological malignancies (Allahyari et al., 2017; Pemmaraju et al., 2019; Shafiee et al., 2019; Kim et al., 2020; Weber, 2015).

Immunotoxin	Market drug name	Binding moiety	Toxin moiety	Bacterial toxin	Disease^a^	Clinical progress
DT388 GM-CSF	--	GM-CSF	DAB388	DT	AML	Phase I
A-dmDT390-bisFv (UCHT1)	--	Anti-CD3	DT390	DT	T-cell-derived malignancies including MF-CTCL	Phase II Completed
DAB486IL2	--	IL-2	DT486	DT	NHL and CTCL	Phase II
DAB389IL2	Denileukin diftitox (Ontak)	IL-2	DAB389	DT	CTCL, NHL, CLL, and more diseases	Accelerated FDA approval in February 1999 and full approval in 2006
HA22	Moxetumomab pasudotox (Lumoxiti)	Anti-CD22 dsFv	PE38	PE	HCL	FDA approved in September of 2018
DT388IL3	Tagraxofusp (Elzonris)	IL-3	DT388	DT	BPDCN	FDA approved in December of 2018

^a^ NHL, non-Hodgkin lymphoma; MF-CTCL, mycosis fungoides-type cutaneous T-cell lymphoma; CTCL, cutaneous T-cell lymphoma; AML, acute myeloid leukemia; CLL, chronic lymphocytic leukemia.

### Moxetumomab pasudotox (Lumoxiti)

MP (HA22) is an FDA-approved RIT targeting CD22 against R/R HCL for adults who previously received ≥2 systemic therapies, including PNA (Kreitman et al., 2012). CD22 is a popular B-cell surface target for RIT against HCL; Olejniczak et al. (2006) utilized flow cytometry and showed that 100% of the nine examined HCL specimens are CD22-positive. Their data also suggest that the CD22 antigen exhibits negligible change following B-cell neoplastic transformation, which makes it a stable targeting marker (Olejniczak et al., 2006). Most importantly, it is not displayed on the B-stem-cell surface (FitzGerald et al., 2004).

The HA22 RIT is composed of anti-CD22 Fv (V_L_ and V_H_ domains), cloned from the murine IgG clone RFB4 (Mansfield et al., 1997), genetically linked to PE38 at V_H_ using recombinant DNA technology, and then produced in *E. coli* (Kreitman et al., 2012). V_L_ and V_H_ in HA22 have three aa substitutions (THW) in place of cysteines (SSY) to increase the high binding affinity and cytotoxicity of RFB4 in the RIT (Kreitman and Pastan, 2011). RFB4 is an excellent binding domain in HA22 due to its high selectivity in binding malignant B-cells with no perceptible evidence of binding normal/benign cells (Mansfield et al., 1997). Additional high-affinity mutants are being tested in a phase-I clinical trial (Salvatore et al., 2002).

### From bench to bedside: milestones to FDA approval

The clinical phase-I (NCT00586924), stage-1 “dose-escalation” cohort included 28 HCL patients treated with MP at three doses ranging from 5 to 50 μg/kg administered every other day to evaluate drug safety. The treatment continued to a maximum of 16 cycles per patient (with ≤4 weeks between cycles) or until disease progression. This stage revealed 86% response rates throughout dose escalation, including 46% with durable CR, with only one case sustaining CR for less than a year. One patient experienced disease progression after the first treatment cycle and was terminated from the study. Of the 27 remaining eligible patients, 3 had stable disease, 13 had CR, and 11 had PR. Immunogenicity assessment of 26 evaluable patients showed 65% with antitoxin-binding Abs after a median of two cycles and 38% developing neutralizing antibodies (nAbs) blocking <75% of 1 μg/mL of IT after at least two cycles; however, among the patients undergoing 5–16 cycles/patient, five patients showed no nAbs. Only one patient had nAbs after the first cycle. Patients with Abs neutralizing <75% of 1 μg/mL of IT qualified for continued treatment as blood levels of the drug were greater than the Ab levels. No dose-limiting responses were reported (Kreitman et al., 2012; Kreitman et al., 2018b).

In stage 2, this phase study was expanded by recruiting 21 additional participants for the 50 μg/kg dose, which was administered in 3 doses over 4-week cycles given every other day. The results showed MRD eradication in the blood and bone marrow in most CR patients, contributing to enduring CR. At a 50 μg/kg dose, the participants’ median duration to reach CR is 42.4 months. The MRD-positive cases of the CR-positive population represented 45% (9 cases), with a median of CR-positive duration of 13.5 months. The MRD-negative population (11 patients) did not reach the median duration of CR. Altogether, this concluded the successful completion of the phase-I trial and unlocked the phase-III clinical trial (Kreitman et al., 2018b).

The phase-III pivotal study (CD-ON-CAT8015-1053) was a third-line, multi-center (in 14 different countries, with the majority of the centers in the United States), open-label efficacy trial. A total of 80 patients were enrolled and treated with 40 μg/kg on days 1, 3, and 5 of every 28-day cycle for a maximum total of six cycles. The results revealed a total of 79% CR and PR, in addition to 80% hematological remissions. Of the population that responded to treatment, 85% tested negative for MRD on immunohistochemistry slides. Overall, the results concluded a durable CR rate along with MRD eradication in R/R HCL patients (Kreitman et al., 2018a). MP was approved by the FDA in September 2018 after successful clinical outcomes (FDA, 2018a).

In November 2022, the manufacturer of MP announced the discontinuation of the product in an official letter to the FDA, citing “very low clinical uptake,” with the discontinuation effective from August 2023. They stated that the decision does not reflect the safety and efficacy of the drug but anticipated that the complexity of specialized administration, the need for patient monitoring, and potential prophylactic toxicity may have affected the observed clinical uptake (FDA, 2023).

### Immunogenicity assessment

An electrochemiluminescent immunoassay was used to assess for the presence of anti-drug antibodies (ADA)—anti-MP. Of the ADA-positive population, nAbs levels were measured using a cell-based assay. A phase-III study (CD-ON-CAT8015-1053) revealed that 59% of the participants were ADA-positive. These ADA-positive patients proceeded to nAb testing, and 95.7% of them had detectable nAbs. Of the nAb-positive cases, 99% exhibited PE38-binding domain-specific ADA, and 54% had CD22-binding domain-specific ADA. If the participants were baseline ADA-positive, they had reduced systemic drug concentrations (FDA, 2018a). Results from the phase-III clinical study reported that the median CD19 B-cell count in the peripheral blood on day 8 declined by 90% and remained reduced throughout the study. Six months after treatment, PR/CR patients exhibited relatively normal B-cell counts (Kreitman et al., 2018a).

### Toxicity and adverse outcomes

Reactions related to infusion account for ≤20% and include diarrhea (≤50%), nausea, edema, headache, anemia, and fever. Renal toxicity and electrolyte alterations were also noted. Other severe effects include capillary leak syndrome (CLS) and hemolytic uremic syndrome (FDA, 2018a).

### Recommended administration protocol: dosing and route.

FDA regulations recommend administering 40 μg/kg through a 30-min intravenous (IV) infusion on days 1, 3, and 5 of each 28-day cycle. The regimen is to advance for a maximum of six cycles, until disease progression or intolerable toxicity.

### Tagraxofusp

Tagraxofusp is an FDA-approved RIT for relapsed or naïve BPDCN, a blood neoplasm that is characterized by IL-3 receptor (CD123) overexpression (El et al., 2020). The RIT is composed of recombinant human IL-3 fused to a truncated DT by a His-Met dipeptide linker and is expressed in *E. coli* (FDA, 2018b). IL-3 binds to the CD123 marker on the tumor cell surface (Economides et al., 2019). IL-3R is a potent target associated with low-to-no myelosuppression susceptibility due to the receptor’s low expression on benign blood cells (Fanny et al., 2013). Tagraxofusp is currently being investigated for post-transplant maintenance of CD123-positive chronic blood malignancies such as AML (clinicaltrial.gov ID: NCT05233618), among other indications summarized in Table 2.

**TABLE 2:** Ongoing clinical trial of Tagraxofusp against blood cancers (National Library of Medicine, 2024a).

Indication/disease^a^	Clinical trial ID
Post-transplant maintenance of CD123-positive chronic blood malignancies: AML, MF, CMML	NCT05233618
CD123-positive or BPDCN-IPh-like AML	NCT04342962
(Tagraxofusp + decitabine) CMML	NCT05038592
(Tagraxofusp + gemtuzumab) R/R AML	NCT05716009
Pediatrics R/R CD123-positive hematological malignancies (ALL, AML, AUL, BPDCN, HL, LL, BCL, TCL, MDS, and MPAL)	NCT05476770

^a^ CMML, chronic myelomonocytic leukemia; MF, myelofibrosis; BPDCN-IPh-like AML, blastic plasmacytoid dendritic cell neoplasm immunophenotype-like acute myeloid leukemia; ALL, acute lymphoblastic leukemia; AUL, acute undifferentiated leukemia; HL, Hodgkin’s lymphoma; LL, lymphoblastic lymphoma; BCL, B-cell lymphoma; TCL, T-cell lymphoma; MDS, myelodysplastic syndrome; MPAL, mixed-phenotype acute leukemia.

### From bench to bedside: milestones to FDA approval

Preliminary pharmacological studies measured the *in vitro* and *in vivo* cytolytic activity. Researchers demonstrated tagraxofusp’s ability to increase apoptosis and reduce cell proliferation using MTT assays and flow cytometry with AV/7AAD staining in model cell lines (CAL-1 and GEN 2.2) and primary BPDCN (collected from 12 patients) cells. They designed various treatment conditions for the MTT assays to assess for toxicity at different concentrations, as monotherapy versus co-administration with other toxins or chemotherapeutics, at 18 h versus 48 h post-treatment. The experimental results of Fanny et al. (2013) reported the notable potency of the drug as a single agent. They confirmed the superior benefit of tagraxofusp to chemotherapy in seven out of eight tested chemotherapeutic agents. Fanny et al. (2013) also showed a reduction in the viability of BPDCN cell lines (92%) and primary cells (80%) at low drug concentrations.

Tagraxofusp showed an increase in survival rate after a single treatment cycle *in vivo*, following intraperitoneal injection into NSG mice 7 days after BPDCN tumor induction (Fanny et al., 2013). Non-clinical toxicology studies were conducted in cynomolgus monkeys to determine the sufficient human-equivalent dose (FDA, 2018b).

To translate these outcomes clinically, a pilot study was conducted for initial clinical validation. Nine participants were evaluable out of 11 recruited. Results revealed a 78% positive response to treatment. A larger multi-center, four-staged, multi-cohort, open-label, single-arm, phase-I/II clinical trial (study STML-401-0114) in adults (≥18 years old), along with three pharmacometrics reports, provided sufficient pharmacological assessment for FDA review.

In the pivotal cohort, 54% of patients entered the CR/complete remission composite (CRc). Notably, no pediatric population was recruited throughout the trial. However, tagraxofusp is indicated for children (≥2 years old) because three pediatric cases treated with the drug showed a biological profile comparable to that observed in adult BPDCN patients (FDA, 2018b).

In STML-401-0114 (clinicaltrial.gov ID: NCT02113982), tagraxofusp was administered for 5 days with a cycle length of 21 days each throughout all four stages of the trial. The results from all stages were interpreted upon reaching the primary clinical endpoint of CR and CRc percentages.

Stage 1 (dose-escalation) aimed to examine the maximum dose tolerance (dose range 7 µg–16 µg per kg of body weight daily for 5 days). Twenty-three patients with BPDCN or refractory/relapsed (R/R) AML were included. The maximum tolerated dose was determined to be 12 µg per kg of body weight daily for 5 days and was used in the subsequent study stages. Therefore, this dose was used in stages 2–4 (FDA, 2018b).

Stage 2 included the same patient criteria, BPDCN or R/R AML, but a larger sample size of 58 cases. A different population was recruited in the “pivotal cohort” stage 3, where 13 patients with untreated newly diagnosed BPDCN were included. In stage 4, referred to as “continued access,” 16 additional participants were enrolled (FDA, 2018b).

One of the centers in the clinical trial included 47 BPDCN patients—32 of whom were untreated, and 15 were relapsed patients. Conclusions from this trial reported a 90% treatment response rate, with the majority achieving CR—59% at 18 months and 52% at 24 months, even in the BPDCN-naïve group. The overall response rate for the refractory group was 67%, with a median overall survival of 8.5 months. Additionally, 45% of the cases advanced to HSCT (Pemmaraju et al., 2019). Tagraxofusp was approved by the FDA for clinical use on December 21, 2018 (Olejniczak et al., 2006; FDA, 2018b; Elzonris, 2020).

Pemmaraju et al. (2022) reported an update in July 2022 on the positive long-term effects of tagraxofusp (with a median follow-up of 34 months), in patients previously enrolled in clinical trials. Overall, rapid treatment response and durable CR + CRc were noted. Results from this recent assessment showed a 75% objective response rate, including 57% CR + CRc. A total of 51% of patients achieving CR + CRc were advanced to either autologous or allogenic stem-cell transplant. The median overall survival of those who achieved CR + CRc and received stem-cell transplants is 38.4 months, and 72% of the patients were in remission for a year or longer after transplant. A total of 37% of patients in CR + CRc who received transplant had major reductions in baseline bone marrow blasts (12%–94%). Furthermore, 4 out of 18 patients with CR + CRc did not undergo transplant, 2 of whom had 27- and 52-month response durations. A novel finding was also reported in the R/R BPDCN patient population, where a 58% response rate was reported after 1–2 treatment cycles (Pemmaraju et al., 2022).

### Immunogenicity assessment

The established vaccination protocol recommends that children (≥6 years old) and infants receive diphtheria vaccination (Centers for Disease Control and Prevention, 2022). In light of this, of the four clinical trials in the tagraxofusp FDA approval summary, pre-existing Abs against DT were detected in 96% of the patients prior to tagraxofusp treatment, 21% of which were neutralizing Abs (nAbs). At the end of cycle two, the antibody titer and frequency of patients with ADA (99%) increased, with 85% having nAbs. After cycle three, 68% of the patients also expressed anti-IL-3 Abs. The study conclusions suggest higher susceptibility to adverse effects in the group with pre-existing Abs at baseline (FDA, 2018b).

### Toxicity and adverse outcomes

CLS was reported in 55% of the participating patients (FDA, 2018b), including several fatalities (Pemmaraju et al., 2019; FDA, 2018b; Elzonris, 2020), and affected 46% as either grade-1 or grade-2 adverse events. Hepatotoxicity was also reported in 40% of the patients. Other chemistry and hematology abnormalities include thrombocytopenia, hypocalcemia, low sodium levels, and hyperglycemia. Hypersensitivity reactions were considered for precautionary measures as they accounted for a 10% incidence rate in the trials (FDA, 2018b).

In the 2022 update by Pemmaraju et al. (2022) on the long-term effects of tagraxofusp, they reported nine grade-5 and three treatment-related CLS events. To resolve these events, tagraxofusp treatment was paused, albumin was administered to all participants with CLS, and steroids were administered to some CLS patients. Nine patients continued receiving additional doses of tagraxofusp after CLS resolution and did not report recurrence (Pemmaraju et al., 2022).

The major hazardous condition of CLS was listed as a boxed warning (Elzonris, 2020). An *in vivo* study using a rat model showed that the prophylactic use of 15-deoxyspergualin (DSG), an inhibitor of NF-kB, reduced vascular leak syndrome when co-administered with a ricin-based IT. Siegall et al. (1994) suggested that prophylactic DSG administration not only allowed for dose escalation that is necessary for successful treatment with IT, but it also did not decline or affect the conjugate’s anti-tumor activity. Further studies may be extended to evaluate whether similar results may be reproduced using DSG with tagraxofusp and other bacterial-based RITs (Siegall et al., 1994). Moreover, severe outcomes during the clinical trial period warranted avid monitoring for dose-modification as needed (Elzonris, 2020; Pemmaraju et al., 2022).

### Recommended administration protocol: dosing and route

The FDA-recommended dosage of tagraxofusp is 12 μg per kg of body weight to be administered intravenously on days 1–5 of a 21-day cycle. The regimen is to continue with repeat cycles until disease progression or unacceptable toxicity is reached (Elzonris, 2020).

### Denileukin diftitox

**FIGURE 2:**
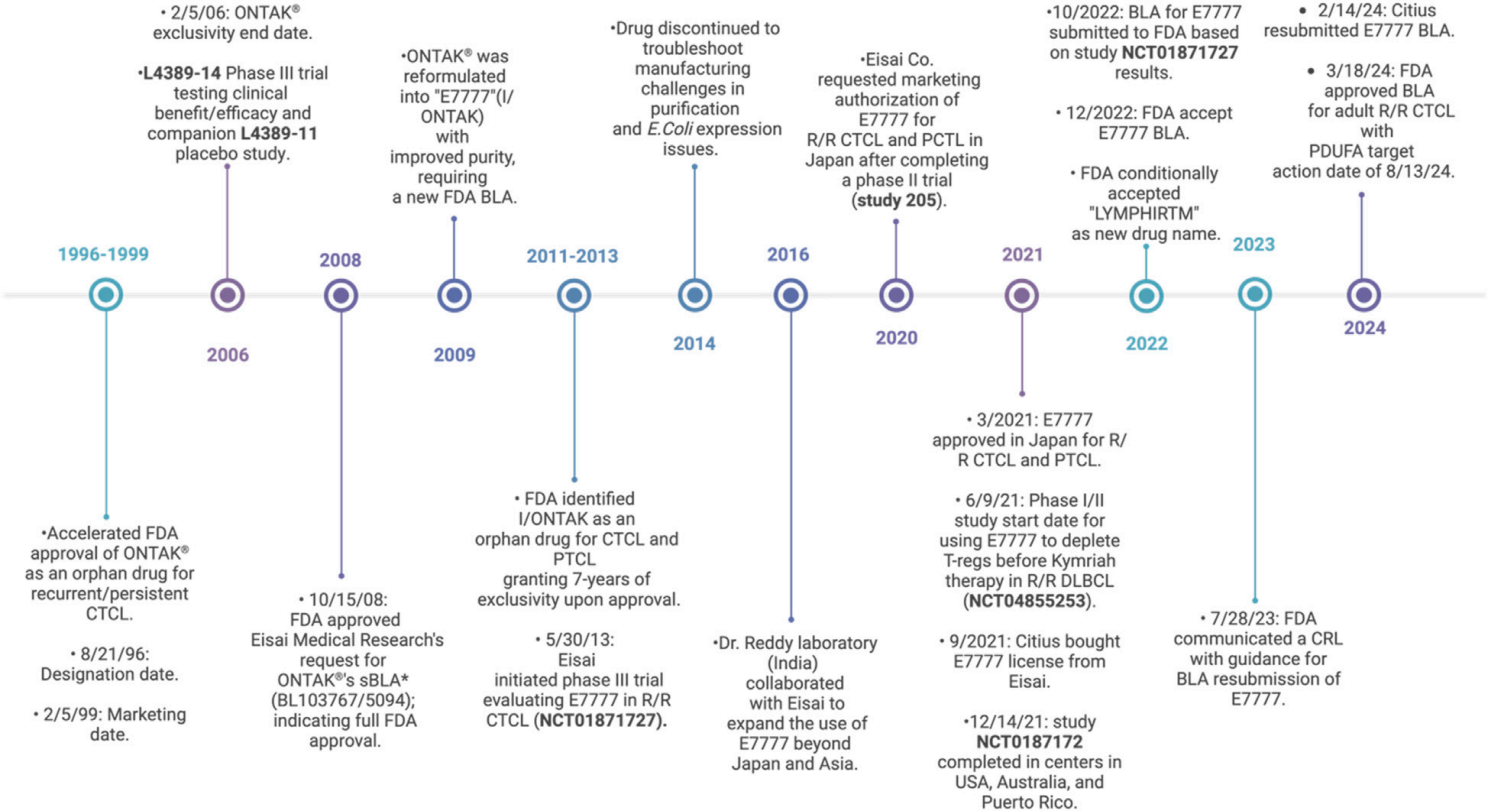
Timeline of clinical status from DD to E7777 (DD-cxdl). Peripheral T-cell lymphoma (PTCL), biologics license application (BLA), supplemental biologics license application (sBLA), complete response letter (CRL), and Prescription Drug User Fee Act (PDUFA) (FDA, 2024; FDA, 2008; Co, 2024; Mahdi et al., 2023; Allen, 2024; LaHucik, 2024; Wang et al., 2017; Eisai, 2024; Shiiba et al., 2022; National Library of Medicine, 2024b; Citiuspharma, 2024; Wrigley, 2022; Conroy, 2022; PR Newswire, 2023; Sava, 2024; Salsburg, 2024).

In 1999, DAB389IL2 (DD) was the first FDA-approved RIT marketed as “Ontak.” In 2009, Ontak was reformulated as E7777 (Lymphir) with further purification to reduce protein aggregates and thereby increase the number of active monomers; however, it retained the same amino acid sequence as DD. In March 2024, the FDA approved E7777 as a new drug for R/R CTCL based on the results from a phase-III multicenter clinical trial, as detailed in Figure 2. E7777 combines human IL-2 with DT and mainly targets IL-2Rα (CD25) on tumor cells. Interestingly, E7777 has also been reported to function against tumors with lower CD25 expression due to its binding to all types of IL-2R, not exclusively CD25 (IL-2Rα). Foss et al. (2022) reported that E7777 showed ∼two times higher specific bioactivity than Ontak (Ghelani et al., 2020).

### From bench to bedside: milestones to FDA approval

Lymphir (E7777) reemerged as a new FDA drug. The approval was supported by the clinical safety and efficacy assessment in NCT01871727 (study 302). Progression-free survival (PFS), quality of life, and time to progression (TTP) were also investigated. The established efficacy endpoints were the objective response rate (ORR) per independent review committee (IRC) based on Global Response Scoring (GRS) as a primary endpoint and the time to response (TTR) and duration of response (DOR) as secondary endpoints. Other endpoints included safety, drug tolerance, and immunogenicity.

The study was conducted in 20 centers across the United States and Australia from May 2013 to December 2021. Two stages were undertaken: the lead-in and the main study. Participants were recruited based on a histopathological CTCL diagnosis and no history of Ontak treatment. Of the 112 recruited patients (median age: 64 years old and average: 62.9 years old) with CTCL stages I–III at baseline, 98 were enrolled and received treatment. Sixty-nine of those patients (five from lead-in and 64 from the main study) plus two additional patients with visceral disease (n = 71) were selected for primary efficacy analysis based on ORR for investigator review. Thirty-four of the 69 patients had previously been treated with ≥1 targeted therapies. Results from this clinical trial showed 6 out of 71 with CR, 24 out of 71 with PR, and 33 out of 71 with stable disease. CR was reported if no detectable evidence of the disease was found, and PR was determined based on disease regression and the absence of newly detected sites.

Approximately 70% of patients who responded to E7777 treatment did so after one to two cycles, with a median TTR of 1.41 months. DOR was 6 or 12 months for 13 and 5 participants, respectively (Foss et al., 2022; Inc. E, 2022).

### Immunogenicity assessment

Immunogenicity was tested based on anti-E7777 and anti-IL2 Abs. The samples were collected on day 1 of cycles 1, 2, 3, and 5. In the lead-in phase (dose escalation study), 100% of the patients were reported to have anti-E7777 Abs; comparably, 37.5%, 87.5%, 100%, and 83.3% of the patients had anti-IL-2 Abs on day 1 of the four cycles. In the main study (objective response rate), 85.7%, 91.7%, and 95.7% of the patients were reported to have anti-E7777 Abs on day 1 of cycles 1, 2, and 3, while only 5.5%, 52.3%, and 88.6% of the patients, respectively, had anti-IL-2 Abs at the start of each cycle (Inc. E, 2022).

In a Japanese phase-II clinical trial, an immunogenicity panel of anti-E7777 and anti-IL2 antibodies was tested for neutralizing activity. The samples were collected pre-treatment; on the first day of cycles 1, 2, 3, 5, and 8; and upon treatment completion. Post-treatment, 74.3% of the patients had positive anti‐E7777 Abs. Anti-IL2 and neutralizing Abs increased from 5.4% to 0% at baseline to over 54.3% and 57.1%, respectively. After administration of E7777 in each cycle, the antibodies against E7777 rapidly cleared the drug from the serum.

### Toxicity and adverse outcomes

Safety assessments in Study 302 were not new to E7777 as they were similar to those reported previously with Ontak. Adverse effects included grade-I/II CLS in some patients; other adverse effects were mild, including ∼28% experiencing either chills, higher ALT levels, or peripheral edema; ∼32% experiencing fatigue; and ∼40% experiencing nausea (Foss et al., 2022).

### Recommended administration protocol: dosing and route

Study 302 conducted dose-escalation analysis in the lead-in phase (6 μg/kg–15 μg/kg) and optimized the recommended dosage of 9 μg/kg/day administered for five consecutive days through an IV infusion over 60 min (±10 min) for up to eight cycles (median of six cycles) or until disease progression or unacceptable toxicity. The cycles were scheduled 21 days apart (Foss et al., 2022; Inc. E, 2022).

## Improvement capacities and risk management

Efforts to reduce immunogenicity are evident throughout the evolution of ITs, such as isolating the catalytic moiety of toxins, utilizing fragments of humanized Abs, and introducing DNA recombination technology. Among the newer mitigation strategies are B- and T-cell epitope mutations, SVP-R, and PEGylation (Mazor et al., 2018a; Mazor et al., 2018b; Zheng et al., 2020).

Since multiple studies have demonstrated that nAbs inhibit RIT function, nullifying B-cell epitopes that are naturally associated with nAbs has been suggested to decrease immunogenicity. Mapping and eliminating B-cell epitopes is a promising strategy (Mazor et al., 2018a). An example of this is LMB-100 (PE24 RIT currently in clinical stages), which is deimmunized from human B-cell epitopes (Mazor et al., 2018b). However, a more cost-effective technique was described to target and eliminate only amino acids with a key role in B-cell responses; this was tested in hopes of mitigating immunogenicity in DT-mediated RITs, where pre-existing ADAs from vaccination are detected (Schmohl et al., 2015).

Similarly, T-cell epitope modification was tested. Unlike B-cells, which naturally undergo affinity maturation, T-cell specificity is not altered upon epitope identification. Thus, elimination of T-cell epitopes is suggested to limit ADA formation (Mazor et al., 2018a). Mazor et al. (2014) studied PE38 and reported the presence of eight main T-cell epitopes. They deleted three epitopes and introduced point mutations in key residues of the remaining five epitopes, resulting in the modified LMB-T18 RIT (targeting CD22). Preclinical trials showed high cytotoxic and antitumor activity and a 93% reduction in T-cell epitopes. Furthermore, no new T-cell epitopes emerged as a result of the mutations. LMB-T18 immunogenicity was also tested against patient sera, and a significant decrease in antibody binding activity was reported (Mazor et al., 2014).

In another study, researchers explored the use of synthetic vaccine particles encapsulating rapamycin (SVP-R) for deimmunization purposes. The efficacy of this strategy was evaluated in an LMB-100 mouse model. Results reported durable effects, specificity, and transferable immune tolerance, preventing ADA and nAbs formation against the RIT. It further showed success in mice with baseline nAbs (Mazor et al., 2018b).

Chemical PEGylation (polyethylene glycol) is a technology that is long-established to improve pharmacokinetics/dynamic capacities in drug delivery in general. In the world of RITs, PEG reduces immunogenicity, improves the short half-life of some RITs, and enhances their cytotoxic efficiency. Zheng et al. (2020) published a study in March 2020, concluding that site-specific PEGylation of anti-mesothelin RITs increases their anti-tumor function and improves the drug’s half-life. As noted with different generations of RITs, mutations to improve and enhance product half-life are paramount because they induce more durable remission that is achieved over a shorter period (Zahaf and Schmidt, 2017).

## Conclusion

Immunotherapy with chimeric bacterial toxins as anti-neoplastic agents is showing clinical promise against hematological malignancies (Johannes and Decaudin, 2005). The RITs use the enzymatic domain of a bacterial toxin and the binding of the antibody to target tumor cells (Greer et al., 2018). Generally, the intracellular toxins from PE and DT have been used because they are extremely effective in mammalian cells (Johannes and Decaudin, 2005; Frankel et al., 2002). Other properties influencing the choice of the toxin include the orientation and structure of the enzymatic domain, purification yields, target antigen expression, and off-target toxicity.

More recently, investigations of RITs targeting solid tumors have gained momentum. Pre-clinically, in a BALB/c mouse model, a study tested E7777 in combination with anti-PD-1 against liver and colon cancer; they reported anti-tumor activity and increased overall survival (Ghelani et al., 2020; Mahdi et al., 2023). In a phase-I clinical trial with LMB-100 against mesothelioma and mesothelin-positive cancers, RIT monotherapy application against solid cancers had dose-limiting toxicity and generated anti-drug antibodies (Hassan et al., 2020). Efforts to evaluate LMB-100 combined with other treatments are under investigation (Skorupan et al., 2024).

As with other immunotherapies, RITs have been more effective in hematological diseases than in solid tumors. Blood cancers usually show milder antibody neutralization against either the toxin or Fv, thereby sustaining higher tolerance for extended treatment sessions. In addition, since blood cancers share the same reservoir of immune cells, RIT access to target cells provides increased immune suppression. Comparably, in solid tumors, RIT dose escalation is limited, secondary to rapid immunogenicity with nAbs forming only weeks after treatment (Pemmaraju et al., 2022). Additionally, usually reduced anti-tumor interaction is observed due to lower tumor penetration into solid tumors, considering their physiological structure. The increase in heterogeneity of solid tumors could also affect RIT treatment efficiency (Pirker, 1988).

RITs have historically encountered challenges within the pharmaceutical industry. Biologics that require complex drug development face difficulties in manufacturing and intellectual property management, which increase production costs. At times, companies have even obtained intellectual property with no intention to implement the drug, effectively abandoning its development (Holzman, 2009). Over the years, these challenges have limited the clinical adoption of RITs.

Other technical issues in RIT development have proven challenging. High levels of purified and active protein can be difficult to achieve. Pharmaceutical companies have explored multiple expression systems from bacteria, yeast, and mammalian cells to cell-free protein synthesis. Although mammalian systems offer advantages, they are costly as their yield is limited (Zhu et al., 2017). Bacterial production can reduce investment and production costs for simple proteins, but it does not add post-translational modifications. For example, in the previously FDA-approved preparations of DD (Ontak), expression in *E. Coli* led to struggles with purifying the active drug from misfolded and aggregated proteins. Difficulties with quality control and production, along with the drug’s safety profile, decreased commercial viability. Increased cost and low demand eventually led to Ontak’s discontinuation (Foss et al., 2022). The refined manufacturing and purification process in the reformulated drug Lymphir (E7777) resulted in higher purity of the active protein monomer with a better safety profile (Kawai et al., 2021).

Additionally, patients could undergo further screening to categorize them with more optimal TSA characteristics, such as low ADA, high TSA expression and density, or low antigen shedding, to ensure favorable outcomes.

Overall, RITs show a growing potential against many hematological malignancies, such as BPDCN, R/R HCL, and R/R CTCL, and can potentially be repurposed for other hematological indications. However, this is a growing field, and researchers continue to investigate other risk management strategies to maximize the benefit from this treatment.

## References

[B1] Abou Dalle I. Ravandi F. (2019). Moxetumomab pasudotox for the treatment of relapsed and/or refractory hairy cell leukemia. Expert Rev. Hematol. 12 (9), 707–714. 10.1080/17474086.2019.1643231 31298972

[B2] Alkharabsheh O. Frankel A. E. (2019). Clinical activity and tolerability of SL-401 (Tagraxofusp): recombinant diphtheria toxin and Interleukin-3 in hematologic malignancies. Biomedicines 7 (1), 6. 10.3390/biomedicines7010006 30621282 PMC6466259

[B3] Allahyari H. Heidari S. Ghamgosha M. Saffarian P. Amani J. (2017). Immunotoxin: a new tool for cancer therapy. Tumour Biol. 39 (2), 1010428317692226. 10.1177/1010428317692226 28218037

[B4] Allen I. (2024). Citius pharmaceuticals completes enrollment in the pivotal phase 3 study of its cancer immunotherapy I/ONTAK for the treatment of cutaneous T-Cell lymphoma. Citius Pharma. Available online at: https://www.sec.gov/Archives/edgar/data/1506251/000121390021063614/ea151726ex99-1_citiuspharrm.htm (Accessed February 25, 2024).

[B5] Allured V. S. Collier R. J. Carroll S. F. McKay D. B. (1986). Structure of exotoxin A of Pseudomonas aeruginosa at 3.0-Angstrom resolution. Proc. Natl. Acad. Sci. U. S. A. 83 (5), 1320–1324. 10.1073/pnas.83.5.1320 3006045 PMC323067

[B6] Amet N. Lee H. F. Shen W. C. (2009). Insertion of the designed helical linker led to increased expression of tf-based fusion proteins. Pharm. Res. 26 (3), 523–528. 10.1007/s11095-008-9767-0 19002568 PMC3121561

[B7] Antignani A. Fitzgerald D. (2013). Immunotoxins: the role of the toxin. Toxins (Basel) 5 (8), 1486–1502. 10.3390/toxins5081486 23965432 PMC3760048

[B8] Arber D. A. Orazi A. Hasserjian R. Thiele J. Borowitz M. J. Le Beau M. M. (2016). The 2016 revision to the world Health Organization classification of myeloid neoplasms and acute leukemia. Blood, J. Am. Soc. Hematol. 127 (20), 2391–2405. 10.1182/blood-2016-03-643544 27069254

[B9] Brown J. G. Entwistle J. Glover N. MacDonald G. C. (2008). Preclinical safety evaluation of immunotoxins. Preclinical safety evaluation of biopharmaceuticals A sciencebased approach to facilitating clinical trials. Hoboken, NJ: John Wiley and Sons, 649–668.

[B10] Cancer Statistics (2024). Cancer statistics. Available online at: https://seer.cancer.gov/statfacts/html/leuks.html (Accessed January, 2024).

[B11] Centers for Disease Control and Prevention (2022). Diphtheria, tetanus, and pertussis vaccine recommendations. Centers for Disease Control and Prevention. Available online at: https://www.cdc.gov/vaccines/vpd/dtap-tdap-td/hcp/recommendations.html.

[B12] Chen X. Zaro J. L. Shen W. C. (2013). Fusion protein linkers: property, design and functionality. Adv. Drug Deliv. Rev. 65 (10), 1357–1369. 10.1016/j.addr.2012.09.039 23026637 PMC3726540

[B13] Citiuspharma (2024). Engineered IL-2-diphtheria toxin fusion protein denileukin diftitox for the treatment of rare forms of cancer. Available online at: https://citiuspharma.com/pipeline/I-ONTAK/default.aspx.

[B14] Co E. G. (2024). FDA grants full approval to ONTAK® (denileukin diftitox) for use in patients with cutaneous T-Cell lymphoma (CTCL). News Release. Available online at: https://www.eisai.com/news/news200856.html (Accessed February 24, 2024).

[B15] Conroy R. (2022). FDA accepts BLA for denileukin diftitox in cutaneous persistent/recurrent T-Cell lymphoma. CancerNetwork. Available online at: https://www.cancernetwork.com/view/fda-accepts-bla-for-denileukin-diftitox-in-cutaneous-persistent-recurrent-t-cell-lymphoma (Accessed February 26, 2024).

[B16] Economides M. P. McCue D. Lane A. A. Pemmaraju N. (2019). Tagraxofusp, the first CD123-targeted therapy and first targeted treatment for blastic plasmacytoid dendritic cell neoplasm. Expert Rev. Clin. Pharmacol. 12 (10), 941–946. 10.1080/17512433.2019.1662297 31465247

[B17] Eisai (2024). Eisai submits marketing authorization application in Japan for anticancer agent denileukin diftitox (genetic recombinant) for cutaneous t-cell lymphoma and peripheral t-cell lymphoma. Available online at: https://www.eisai.com/news/2020/pdf/enews202015pdf.pdf (Accessed February 25, 2024).

[B18] El A. H. Dupont E. Paul S. Khoury J. D. (2020). CD123 as a biomarker in hematolymphoid malignancies: principles of detection and targeted therapies. Cancers (Basel) 12 (11), 3087. 10.3390/cancers12113087 33113953 PMC7690688

[B19] Else M. Dearden C. E. Matutes E. Garcia-Talavera J. Rohatiner A. Z. S. Johnson S. A. N. (2009). Long-term follow-up of 233 patients with hairy cell leukaemia, treated initially with pentostatin or cladribine, at a median of 16 years from diagnosis. Br. J. Haematol. 145 (6), 733–740. 10.1111/j.1365-2141.2009.07668.x 19344416

[B20] Elzonris (2020). The largest prospective BPDCN study (N=84) of treatment-naive and previously treated patients. Elzonris. Available online at: https://elzonris.com/hcp/study-design.

[B21] Fanny D. Frankel A. E. Seilles E. Biichle S. Deconninck E. Bonnefoy F. (2013). Preclinical studies of SL-401, a targeted therapy directed to the interleukin-3 receptor (IL3-R), in blastic plasmacytoid dendritic cell neoplasm (BPDCN): potent activity in BPDCN cell lines, primary tumor, and in an *in vivo* model. Blood 122 (21), 3942. 10.1182/blood.V122.21.3942.3942

[B22] Farkona S. Diamandis E. P. Blasutig I. M. (2016). Cancer immunotherapy: the beginning of the end of cancer? BMC Med. 14, 73. 10.1186/s12916-016-0623-5 27151159 PMC4858828

[B23] FDA (2008). Letter response to BLA. Available online at: https://www.accessdata.fda.gov/drugsatfda_docs/appletter/2008/103767s5094ltr.pdf (Accessed February 28th, 2024).

[B24] FDA (2018a). FDA highlights of lumoxiti prescribing information. Available online at: https://www.accessdata.fda.gov/drugsatfda_docs/label/2018/761104s000lbl.pdf.

[B25] FDA (2018b). Tagraxofusp-erzs FDA approval summary review. Available online at: https://www.accessdata.fda.gov/drugsatfda_docs/nda/2018/761116Orig1s000SumR.pdf.

[B26] FDA (2020). FDA approves moxetumomab pasudotox-tdfk for hairy cell leukemia. Available online at: https://www.fda.gov/drugs/resources-information-approved-drugs/fda-approves-moxetumomab-pasudotox-tdfk-hairy-cell-leukemia (Accessed April, 2020).

[B27] FDA (2023). AstraZeneca’s letter to the FDA about discontinuing lumoxiti. Lett. Novemb. 18th. Available online at: https://www.fda.gov/media/164425/download (Accessed February 7, 2024).

[B28] FDA (2024). FDA. Available online at: https://www.accessdata.fda.gov/scripts/opdlisting/oopd/detailedIndex.cfm?cfgridkey=99396 (Accessed February 28th, 2024).

[B29] FitzGerald D. J. Kreitman R. Wilson W. Squires D. Pastan I. (2004). Recombinant immunotoxins for treating cancer. Int. J. Med. Microbiol. 293 (7-8), 577–582. 10.1078/1438-4221-00302 15149034

[B30] Foss F. M. Kim Y. H. Prince H. Kuzel T. M. Yannakou C. K. Ooi C. E. (2022). Efficacy and safety of E7777 (improved purity denileukin diftitox [ONTAK]) in patients with relapsed or refractory cutaneous T-cell lymphoma: results from pivotal study 302. Blood 140 (Suppl. 1), 1491–1492. 10.1182/blood-2022-166916

[B31] Frankel A. E. Rossi P. Kuzel T. M. Foss F. (2002). Diphtheria fusion protein therapy of chemoresistant malignancies. Curr. Cancer Drug Targets 2 (1), 19–36. 10.2174/1568009023333944 12188918

[B32] George R. A. Heringa J. (2002). An analysis of protein domain linkers: their classification and role in protein folding. Protein Eng. 15 (11), 871–879. 10.1093/protein/15.11.871 12538906

[B33] Getta B. M. Park J. H. Tallman M. S. (2015). Hairy cell leukemia: past, present and future. Best. Pract. Res. Clin. Haematol. 28 (4), 269–272. 10.1016/j.beha.2015.10.015 26614906 PMC5008915

[B34] Getta B. M. Woo K. M. Devlin S. Park J. H. Abdel-Wahab O. Saven A. (2016). Treatment outcomes and secondary cancer incidence in young patients with hairy cell leukaemia. Br. J. Haematol. 175 (3), 402–409. 10.1111/bjh.14207 27351754 PMC5539949

[B35] Ghelani A. Bates D. Conner K. Wu M. Z. Lu J. Hu Y. L. (2020). Defining the threshold IL-2 signal required for induction of selective Treg cell responses using engineered IL-2 muteins. Front. Immunol. 11, 1106. 10.3389/fimmu.2020.01106 32582190 PMC7291599

[B36] Greer J. P. Arber D. A. Glader B. E. List A. F. Means R. M. Rodgers G. M. (2018). Wintrobe's clinical hematology. Lippincott Williams and Wilkins.

[B37] Hamadani M. Abu Kar S. M. Usmani S. Z. Savani B. N. Ayala E. Kharfan-Dabaja M. A. (2014). Management of relapses after hematopoietic cell transplantation in T-cell Non-Hodgkin lymphomas. Semin. Hematol. 51 (1), 73–86. 10.1053/j.seminhematol.2013.11.005 24468319

[B38] Hansen J. K. Weldon J. E. Xiang L. Beers R. Onda M. Pastan I. (2010). A recombinant immunotoxin targeting CD22 with low immunogenicity, low nonspecific toxicity, and high antitumor activity in mice. J. Immunother. 33 (3), 297–304. 10.1097/CJI.0b013e3181cd1164 20445350 PMC7291874

[B39] Hassan R. Alewine C. Mian I. Spreafico A. Siu L. L. Gomez-Roca C. (2020). Phase 1 study of the immunotoxin LMB-100 in patients with mesothelioma and other solid tumors expressing mesothelin. Cancer 126 (22), 4936–4947. 10.1002/cncr.33145 32870522 PMC8552963

[B40] Holzman D. C. (2009). Whatever happened to immunotoxins? Research, and hope, are still alive. J. Natl. Cancer Inst. 101 (9), 624–625. 10.1093/jnci/djp110 19401548

[B41] Inc. E (2022). A trial of E7777 in persistent and recurrent cutaneous T-Cell lymphoma. Available online at: https://clinicaltrials.gov/study/NCT01871727 (Accessed June 12, 2024).

[B42] Johannes L. Decaudin D. (2005). Protein toxins: intracellular trafficking for targeted therapy. Gene Ther. 12 (18), 1360–1368. 10.1038/sj.gt.3302557 15902276

[B43] John R. M. (1996). “Corynebacterium Diphtheriae,” in Medical microbiology. Editor B S. 4th ed.

[B44] Kawai H. Ando K. Maruyama D. Yamamoto K. Kiyohara E. Terui Y. (2021). Phase II study of E7777 in Japanese patients with relapsed/refractory peripheral and cutaneous T-cell lymphoma. Cancer Sci. 112 (6), 2426–2435. 10.1111/cas.14906 33792128 PMC8177793

[B45] Khan S. Sawas A. (2019). Antibody-Directed therapies: toward a durable and tolerable treatment platform for CTCL. Front. Oncol. 9, 645. 10.3389/fonc.2019.00645 31417860 PMC6683760

[B46] Kharfan-Dabaja M. A. Lazarus H. M. Nishihori T. Mahfouz R. A. Hamadani M. (2013). Diagnostic and therapeutic advances in blastic plasmacytoid dendritic cell neoplasm: a focus on hematopoietic cell transplantation. Biol. Blood Marrow Transpl. 19 (7), 1006–1012. 10.1016/j.bbmt.2013.01.027 23396213

[B47] Kim J. S. Jun S. Y. Kim Y. S. (2020). Critical issues in the development of immunotoxins for anticancer therapy. J. Pharm. Sci. 109 (1), 104–115. 10.1016/j.xphs.2019.10.037 31669121

[B48] Kolybo D. Labyntsev A. Korotkevich N. Komisarenko S. Romaniuk S. Oliinyk O. (2013). Immunobiology of diphtheria. Recent approaches for the prevention, diagnosis, and treatment of disease. Biotechnol. Acta 6 (4), 043–062. 10.15407/biotech6.04.043

[B49] Kreitman R. J. Pastan I. (2011). Antibody fusion proteins: anti-CD22 recombinant immunotoxin moxetumomab pasudotox. Clin. Cancer Res. 17 (20), 6398–6405. 10.1158/1078-0432.CCR-11-0487 22003067 PMC3201735

[B50] Kreitman R. J. Pastan I. (2020). Development of recombinant immunotoxins for hairy cell leukemia. Biomolecules 10 (8), 1140. 10.3390/biom10081140 32756468 PMC7464581

[B51] Kreitman R. J. Tallman M. S. Robak T. Coutre S. Wilson W. H. Stetler-Stevenson M. (2012). Phase I trial of anti-CD22 recombinant immunotoxin moxetumomab pasudotox (CAT-8015 or HA22) in patients with hairy cell leukemia. J. Clin. Oncol. 30 (15), 1822–1828. 10.1200/JCO.2011.38.1756 22355053 PMC3383181

[B52] Kreitman R. J. Dearden C. Zinzani P. L. Delgado J. Karlin L. Robak T. (2018a). Moxetumomab pasudotox in relapsed/refractory hairy cell leukemia. Leukemia 32 (8), 1768–1777. 10.1038/s41375-018-0210-1 30030507 PMC6087717

[B53] Kreitman R. J. Tallman M. S. Robak T. Coutre S. Wilson W. H. Stetler-Stevenson M. (2018b). Minimal residual hairy cell leukemia eradication with moxetumomab pasudotox: phase 1 results and long-term follow-up. Blood, J. Am. Soc. Hematol. 131 (21), 2331–2334. 10.1182/blood-2017-09-803072 29487070 PMC5969375

[B54] Kreitman R. J. Dearden C. Zinzani P. L. Delgado J. Robak T. le Coutre P. D. (2021). Moxetumomab pasudotox in heavily pre-treated patients with relapsed/refractory hairy cell leukemia (HCL): long-term follow-up from the pivotal trial. J. Hematol. Oncol. 14 (1), 35. 10.1186/s13045-020-01004-y 33627164 PMC7905554

[B55] Krolick K. A. Uhr J. W. Slavin S. Vitetta E. S. (1982). *In vivo* therapy of a murine B cell tumor (BCL1) using antibody-ricin A chain immunotoxins. J. Exp. Med. 155 (6), 1797–1809. 10.1084/jem.155.6.1797 6804591 PMC2186702

[B56] LaHucik K. (2024). Citius buys out license to Ontak replacement from dr. Reddy's for $40M. Available online at: https://www.fiercepharma.com/drug-delivery/citius-buys-out-license-to-ontak-replacement-from-dr-reddy-s-for-40m (Accessed February 24, 2024).

[B57] Mahdi H. S. Woodall-Jappe M. Singh P. Czuczman M. S. (2023). Targeting regulatory T cells by E7777 enhances CD8 T-cell–mediated anti-tumor activity and extends survival benefit of anti-PD-1 in solid tumor models. Front. Immunol. 14, 1268979. 10.3389/fimmu.2023.1268979 38022532 PMC10646188

[B58] Maitre E. Cornet E. Troussard X. (2019). Hairy cell leukemia: 2020 update on diagnosis, risk stratification, and treatment. Am. J. Hematol. 94 (12), 1413–1422. 10.1002/ajh.25653 31591741

[B59] Mansfield E. Amlot P. Pastan I. FitzGerald D. J. (1997). Recombinant RFB4 immunotoxins exhibit potent cytotoxic activity for CD22-bearing cells and tumors. Blood 90 (5), 2020–2026. 10.1182/blood.v90.5.2020 9292538

[B60] Matthey B. Engert A. Barth S. (2000). Recombinant immunotoxins for the treatment of Hodgkin's disease. Int. J. Mol. Med. 6 (5), 509–523. 10.3892/ijmm.6.5.509 11029515

[B61] Mazor R. Pastan I. (2020). Immunogenicity of immunotoxins containing pseudomonas Exotoxin A: causes, consequences, and mitigation. Front. Immunol. 11, 1261. 10.3389/fimmu.2020.01261 32695104 PMC7333791

[B62] Mazor R. Eberle J. A. Hu X. Vassall A. N. Onda M. Beers R. (2014). Recombinant immunotoxin for cancer treatment with low immunogenicity by identification and silencing of human T-cell epitopes. Proc. Natl. Acad. Sci. U. S. A. 111 (23), 8571–8576. 10.1073/pnas.1405153111 24799704 PMC4060717

[B63] Mazor R. King E. M. Pastan I. (2018a). Strategies to reduce the immunogenicity of recombinant immunotoxins. Am. J. Pathol. 188 (8), 1736–1743. 10.1016/j.ajpath.2018.04.016 29870741 PMC6099333

[B64] Mazor R. King E. M. Onda M. Cuburu N. Addissie S. Crown D. (2018b). Tolerogenic nanoparticles restore the antitumor activity of recombinant immunotoxins by mitigating immunogenicity. Proc. Natl. Acad. Sci. U. S. A. 115 (4), E733–E742. 10.1073/pnas.1717063115 29311317 PMC5789939

[B65] McCarthy E. F. (2006). The toxins of William B. Coley and the treatment of bone and soft-tissue sarcomas. Iowa Orthop. J. 26, 154–158. 16789469 PMC1888599

[B66] Mei X. Chen J. Wang J. Zhu J. (2019). Immunotoxins: targeted toxin delivery for cancer therapy. Pharm. Fronts 1 (01), e33–e45. 10.1055/s-0039-1700507

[B67] Michael Lord J. Roberts L. M. (1998). Toxin entry: retrograde transport through the secretory pathway. J. cell Biol. 140 (4), 733–736. 10.1083/jcb.140.4.733 9472027 PMC2141750

[B68] Michalska M. Wolf P. (2015). Pseudomonas Exotoxin A: optimized by evolution for effective killing. Front. Microbiol. 6, 963. 10.3389/fmicb.2015.00963 26441897 PMC4584936

[B69] Moolten F. L. Capparell N. J. Zajdel S. H. Cooperband S. R. (1975). Antitumor effects of antibody-diphtheria toxin conjugates. II. Immunotherapy with conjugates directed against tumor antigens induced by simian virus 40. J. Natl. Cancer Inst. 55 (2), 473–477. 10.1093/jnci/55.2.473 169378

[B70] Morton L. M. Wang S. S. Devesa S. S. Hartge P. Weisenburger D. D. Linet M. S. (2006). Lymphoma incidence patterns by WHO subtype in the United States, 1992-2001. Blood 107 (1), 265–276. 10.1182/blood-2005-06-2508 16150940 PMC1895348

[B71] Murphy J. R. (2011). Mechanism of diphtheria toxin catalytic domain delivery to the eukaryotic cell cytosol and the cellular factors that directly participate in the process. Toxins 3 (3), 294–308. 10.3390/toxins3030294 22069710 PMC3202816

[B72] National Library of Medicine (2024a). Accessed February 2024. Available online at: https://www.clinicaltrials.gov/search?intr=tagraxofusp&page=1.

[B73] National Library of Medicine (2024b). A trial of E7777 in persistent and recurrent cutaneous T-Cell lymphoma. Available online at: https://www.clinicaltrials.gov/study/NCT01871727?intr=NCT01871727&rank=1 (Accessed February 25, 2024).

[B74] Olejniczak S. H. Stewart C. C. Donohue K. Czuczman M. S. (2006). A quantitative exploration of surface antigen expression in common B-cell malignancies using flow cytometry. Immunol. Invest 35 (1), 93–114. 10.1080/08820130500496878 16531332

[B75] Pastan I. Chaudhary V. FitzGerald D. J. (1992). Recombinant toxins as novel therapeutic agents. Annu. Rev. Biochem. 61, 331–354. 10.1146/annurev.bi.61.070192.001555 1497314

[B76] Pastan I. Hassan R. Fitzgerald D. J. Kreitman R. J. (2006). Immunotoxin therapy of cancer. Nat. Rev. Cancer 6 (7), 559–565. 10.1038/nrc1891 16794638

[B77] Pemmaraju N. Lane A. A. Sweet K. L. Stein A. S. Vasu S. Blum W. (2019). Tagraxofusp in blastic plasmacytoid dendritic-cell neoplasm. N. Engl. J. Med. 380 (17), 1628–1637. 10.1056/NEJMoa1815105 31018069

[B78] Pemmaraju N. Sweet K. L. Stein A. S. Wang E. S. Rizzieri D. A. Vasu S. (2022). Long-Term benefits of tagraxofusp for patients with blastic plasmacytoid dendritic cell neoplasm. J. Clin. Oncol. 40 (26), 3032–3036. 10.1200/JCO.22.00034 35820082 PMC9462530

[B79] Pirker R. (1988). Immunotoxins against solid tumors. J. Cancer Res. Clin. Oncol. 114 (4), 385–393. 10.1007/BF02128183 3045130 PMC12243822

[B80] PR Newswire (2023). Citius pharmaceuticals, Inc. Receives regulatory guidance from the U.S. food and Drug Administration (FDA) regarding the planned resubmission of the BLA for LYMPHIR™. Citius Pharmaceuticals, Inc. Available online at: https://www.prnewswire.com/news-releases/citius-pharmaceuticals-inc-receives-regulatory-guidance-from-the-us-food-and-drug-administration-fda-regarding-the-planned-resubmission-of-the-bla-for-lymphir-301921591.html (Accessed February 26, 2024).

[B81] Rauh M. J. Rahman F. Good D. Silverman J. Brennan M. K. Dimov N. (2012). Blastic plasmacytoid dendritic cell neoplasm with leukemic presentation, lacking cutaneous involvement: case series and literature review. Leuk. Res. 36 (1), 81–86. 10.1016/j.leukres.2011.07.033 21890199

[B83] Salsburg G. (2024). Citius pharmaceuticals announces FDA acceptance of the BLA resubmission of LYMPHIR™ (denileukin diftitox) for the treatment of adults with relapsed or refractory cutaneous T-Cell lymphoma. Citius Pharma News Available online at: https://citiuspharma.com/investors/news-media/news/release-details/2024/Citius-Pharmaceuticals-Announces-FDA-Acceptance-of-the-BLA-Resubmission-of-LYMPHIR-Denileukin-Diftitox-for-the-Treatment-of-Adults-with-Relapsed-or-Refractory-Cutaneous-T-Cell-Lymphoma/default.aspx (Accessed August 23, 2024).

[B84] Salvatore G. Beers R. Margulies I. Kreitman R. J. Pastan I. (2002). Improved cytotoxic activity toward cell lines and fresh leukemia cells of a mutant anti-CD22 immunotoxin obtained by antibody phage display. Clin. Cancer Res. 8 (4), 995–1002. 10.1158/1078-0432.CCR-01-0450 11948105

[B85] Sava J. (2024). Denileukin diftitox BLA resubmitted for CTCL after addressing FDA concerns. Available online at: https://www.targetedonc.com/view/denileukin-diftitox-bla-resubmitted-for-ctcl-after-addressing-fda-concerns (Accessed February 26, 2024).

[B86] Schmohl J. U. Todhunter D. Oh S. Vallera D. A. (2015). Mutagenic deimmunization of diphtheria toxin for use in biologic drug development. Toxins (Basel) 7 (10), 4067–4082. 10.3390/toxins7104067 26473923 PMC4626721

[B87] Seymour J. F. Kurzrock R. Freireich E. J. Estey E. H. (1994). 2-chlorodeoxyadenosine induces durable remissions and prolonged suppression of CD4+ lymphocyte counts in patients with hairy cell leukemia. Blood 83 (10), 2906–2911. 10.1182/blood.v83.10.2906.2906 7910051

[B88] Seymour J. F. Talpaz M. Kurzrock R. (1997). Response duration and recovery of CD4+ lymphocytes following deoxycoformycin in interferon-alpha-resistant hairy cell leukemia: 7-year follow-up. Leukemia 11 (1), 42–47. 10.1038/sj.leu.2400513 9001417

[B89] Shafiee F. Aucoin M. G. Jahanian-Najafabadi A. (2019). Targeted diphtheria toxin-based therapy: a review article. Front. Microbiol. 10, 2340. 10.3389/fmicb.2019.02340 31681205 PMC6813239

[B90] Shi Y. Wang E. (2014). Blastic plasmacytoid dendritic cell neoplasm: a clinicopathologic review. Arch. Pathol. Lab. Med. 138 (4), 564–569. 10.5858/arpa.2013-0101-RS 24678689

[B91] Shiiba H. Takechi A. Asakura S. Kawaguchi T. Sato M. (2022). Preclinical and clinical researches of Denileukin Diftitox (genetical Recombination)(Remitoro®), a novel agent for T-cell lymphoma. Nihon Yakurigaku zasshi Folia Pharmacol. Jpn. 157 (5), 376–382. 10.1254/fpj.22032 36047157

[B92] Shimony S. Luskin M. R. Gangat N. LeBoeuf N. R. Feraco A. M. Lane A. A. (2025). Blastic Plasmacytoid Dendritic Cell Neoplasm (BPDCN): 2025 update on diagnosis, pathophysiology, risk assessment, and management. Am. J. Hematol. 100 (8), 1408–1422. 10.1002/ajh.27737 40525728

[B93] Siegall C. B. Liggitt D. Chace D. Tepper M. A. Fell H. P. (1994). Prevention of immunotoxin-mediated vascular leak syndrome in rats with retention of antitumor activity. Proc. Natl. Acad. Sci. U. S. A. 91 (20), 9514–9518. 10.1073/pnas.91.20.9514 7937798 PMC44843

[B94] Skorupan N. Peer C. J. Zhang X. Choo-Wosoba H. Ahmad M. I. Lee M. J. (2024). Tofacitinib to prevent anti-drug antibody formation against LMB-100 immunotoxin in patients with advanced mesothelin-expressing cancers. Front. Oncol. 14, 1386190. 10.3389/fonc.2024.1386190 38706610 PMC11066227

[B95] Srivastava S. Luqman S. (2015). Immune-O-Toxins as the magic bullet for therapeutic purposes. Biomed. Res. Ther. 2 (1), 2–15. 10.7603/s40730-015-0002-4

[B96] Tadmor T. (2011). Purine analog toxicity in patients with hairy cell leukemia. Leuk. Lymphoma 52 (Suppl. 2), 38–42. 10.3109/10428194.2011.565097 21463124

[B97] Tay J. Daly A. Jamani K. Labelle L. Savoie L. Stewart D. (2019). Patient eligibility for hematopoietic stem cell transplantation: a review of patient-associated variables. Bone Marrow Transpl. 54 (3), 368–382. 10.1038/s41409-018-0265-7 29988063

[B98] Valent P. Groner B. Schumacher U. Superti-Furga G. Busslinger M. Kralovics R. (2016). Paul Ehrlich (1854-1915) and his contributions to the foundation and birth of translational medicine. J. Innate Immun. 8 (2), 111–120. 10.1159/000443526 26845587 PMC6738855

[B99] Wang Z. Zheng Q. Zhang H. Bronson R. T. Madsen J. C. Sachs D. H. (2017). Ontak-like human IL-2 fusion toxin. J. Immunol. Methods 448, 51–58. 10.1016/j.jim.2017.05.008 28551309 PMC5576150

[B100] Weber G. F. (2015). Molecular therapies of cancer. Springer.

[B101] Weldon J. E. Pastan I. (2011). A guide to taming a toxin--recombinant immunotoxins constructed from Pseudomonas exotoxin A for the treatment of cancer. FEBS J. 278 (23), 4683–4700. 10.1111/j.1742-4658.2011.08182.x 21585657 PMC3179548

[B102] Weldon J. E. Skarzynski M. Therres J. A. Ostovitz J. R. Zhou H. Kreitman R. J. (2015). Designing the furin-cleavable linker in recombinant immunotoxins based on Pseudomonas exotoxin A. Bioconjug Chem. 26 (6), 1120–1128. 10.1021/acs.bioconjchem.5b00190 25997032 PMC7724502

[B103] Wolf P. Elsasser-Beile U. (2009). Pseudomonas exotoxin A: from virulence factor to anti-cancer agent. Int. J. Med. Microbiol. 299 (3), 161–176. 10.1016/j.ijmm.2008.08.003 18948059

[B104] Wrigley N. (2022). BLA submitted to the FDA for denileukin diftitox in cutaneous Persistent/Recurrent T-Cell lymphoma. CancerNetwork. Available online at: https://www.cancernetwork.com/view/bla-submitted-to-the-fda-for-denileukin-diftitox-in-cutaneous-persistent-recurrent-t-cell-lymphoma (Accessed February 25, 2024).

[B105] Yamaizumi M. Mekada E. Uchida T. Okada Y. (1978). One molecule of diphtheria toxin fragment A introduced into a cell can kill the cell. Cell 15 (1), 245–250. 10.1016/0092-8674(78)90099-5 699044

[B106] Yeung C. C. S. Radich J. (2017). Predicting chemotherapy resistance in AML. Curr. Hematol. Malig. Rep. 12 (6), 530–536. 10.1007/s11899-017-0378-x 28397032

[B107] Yu S. Kwon M. J. Kim K. Koo D. H. Woo H. Y. Park H. (2014). A rare case of acute leukemic presentation of blastic plasmacytoid dendritic cell neoplasm without cutaneous lesions. Ann. Lab. Med. 34 (2), 148–151. 10.3343/alm.2014.34.2.148 24624352 PMC3948829

[B108] Zahaf N. I. Schmidt G. (2017). Bacterial toxins for cancer therapy. Toxins (Basel) 9 (8), 236. 10.3390/toxins9080236 28788054 PMC5577570

[B109] Zdanovsky A. G. Chiron M. Pastan I. FitzGerald D. J. (1993). Mechanism of action of Pseudomonas exotoxin. Identification of a rate-limiting step. J. Biol. Chem. 268 (29), 21791–21799. 10.1016/s0021-9258(20)80612-7 8408034

[B110] Zheng Z. Okada R. Kobayashi H. Nagaya T. Wei J. Zhou Q. (2020). Site-Specific PEGylation of Anti-Mesothelin recombinant immunotoxins increases half-life and antitumor activity. Mol. Cancer Ther. 19 (3), 812–821. 10.1158/1535-7163.MCT-19-0890 31871266 PMC7056543

[B111] Zhu S. Liu Y. Wang P. C. Gu X. Shan L. (2017). Recombinant immunotoxin therapy of glioblastoma: smart design, key findings, and specific challenges. Biomed. Res. Int. 2017, 7929286. 10.1155/2017/7929286 28752098 PMC5511670

